# Midwifery

**Published:** 1826-01

**Authors:** 


### MIDWIFERY.

1. *Secale Cornutum, or Ergot of Rye.** This very curious vegetable production has excited much more attention in America than in this country. Dr. Davies has now laid before the public all the information in his power respecting it—partly from his own observation, partly from that of his friends. The substance in question is known to be a disease, in which one or more of the grains of rye are usually enlarged or elongated, projecting from the spike or pannicle to which they belong, and being of an irregular curved form, somewhat resembling the spur of a cock—of a light brittle texture, dark colour, and unpleasant smell. The cause of this diseased production in rye is not satisfactorily accounted for. Various maladies are said to have been occasioned by feeding on bread made of this diseased rye, as dry gangrene, and other affections. Dr. Stearns, of New York, appears to have been the first who called the attention of the profession to this peculiar article, as possessing great power of exciting the action of the gravid uterus. The testimonies in its favour are so numerous that they claim a trial of its effects in this country. "When exhibited in large doses, it produces generally one long-continued action of the womb, which has been known to last for an hour or more, until its contents are expelled. When given prematurely, or under improper circumstances, it has occasionally proved injurious to the mother, and more frequently so to the child; but in moderate doses, at short intervals, it increases the vigour of the pains, without producing such excessive and constant action as may become dangerous." 3. About half a century ago, the midwives in America were in the habit of giving *blasted rye tea* (infusion of ergot) for the purpose of quickening labour, and to make the patient bear down, thus accelerating the birth. Where the presentation was natural and the pains inefficient, the ergot generally produced the evolution of the fœtus in an hour. Where the presentation was unnatural and the parts rigid, it was prejudicial, exciting, under such circumstances, delirium and hysteria. The midwives, observing this, laid aside the medicine, and thus it fell into disuse for want of discrimination. Dr. Stearns again introduced it. Dr. Davies has used this substance in several instances, as have some of his medical friends—from which he is enabled to speak positively of its powers in cases of protracted labour. But he is not able to give such satisfactory evidence of its effects in some uterine diseases as other writers would lead us to expect. In regard to its preparation and exhibition, it may be used either as a powder or infusion—the latter is said to extract all its most active properties. The powder is more speedily administered, but more apt to occasion nausea. The infusion is of a violet colour, nauseous taste, and slightly acrimonious, occasioning, when given in large doses, much heat and pain in

* Dr. Henry Davies. Med. and Phys. Journal, July and August, 1825.

the hypogastric region—sometimes vomiting. Dr. Davies has usually given the powder in doses of a scruple—" or the infusion of one drachm to three ounces of boiling water." The effect has generally been, that, in the space of from three to fifteen minutes after its exhibition, the uterus was excited to strong and violent action, accompanied by sympathetic effects on the neighbouring parts, which pains have continued, with little or no intermission, for a longer or shorter space of time, and then entirely ceased. The effects of the medicine appeared to be solely confined to the uterus and neighbouring parts, the circulation never being hurried, or at all affected.

*Cases.* The first case introduced by our author, is one from Dr. Merriman's work on difficult parturition. It was a twin case; the first child being expelled, and no efforts being made by the uterus to discharge the other. Fifteen hours from the first birth, the infusion of ergot was given, (ʒj. in a tea-cupful of boiling water) and, in fifteen minutes, was followed by uterine action and the protrusion of the child. The child was living and did well.

*Case* 2. *By the same.* In a third labour the waters broke, and after 36 hours the pains ceased, the child's head being situated at the brim of the pelvis. The infusion, as above, was given, and in five minutes the pains came on, and soon ended in delivery.

*Case* 3. Dr. Davies was called to a woman who had been in labour more than 36 hours. She was now free from pain, but unable to lie down. The os uteri was fully dilated, the head firmly wedged in the brim of the pelvis, lying transversely, presenting a puffy tumour with a smooth surface. Dr. D. gave her the infusion of ergot, and in five minutes afterwards she had a sharp pain. In half an hour the child was expelled, and in another half hour the placenta.

*Case* 4. Mrs. Smith, with her first child. Had been in labour two days. The pains were now frequent, feeble, and of no avail. The head of the child was lying high at the brim of the pelvis—the membranes not ruptured—the os uteri not completely effaced. Dr. D. ruptured the membranes, but, at the end of two hours, no advance of the head had taken place. The infusion of ergot was given. It had no effect for 20 minutes afterwards. There was then a slight pain, with inclination to stool. In 40 minutes another dose was given. The pains continued—the head made some progress, but it was extremely slow, and the patient was much exhausted. The forceps were applied, and the delivery of a dead child effected.

*Cases* 5 *and* 6. One by Mr. Hallion, the other by Dr. Davies himself. In both instances, the ergot brought on pains, and effected parturition in a very short time after its exhibition. The same may be said of many other cases related by our author.

From the above, and many other cases published by this intelligent physician, it is evident that the secale possesses peculiar powers in influencing the womb in certain states, "and the effect," says he, "in these states, is as much to be relied on as the action of emetics and purgatives, in cases where they may be required." The administration of the medicine requires precaution, of course, and discrimination, otherwise it may do harm instead of good. The powers of the secale, Dr. D. observes, appear to be wholly confined to its action on the uterine fibres, when that viscus is in a state of enlargement. "It does not appear, however, to bring on labour or cause action in the first instance, but to increase the action, when weak, or renew it when suspended." The healthy unimpregnated uterus is not affected by this substance. In no case has its use been followed by hæmorrhage—nay, it has been said to be useful in uterine hæmorrhage from other causes. The following are our author's directions for its use.

"The secale ought never to be given where there is any natural defect, either in the pelvis or soft parts, capable of producing a powerful obstacle to the expulsion of the child. Neither in those cases where the neck of the uterus is hard, swollen, or painful; in short, where there is rigidity of the parts: and, generally, where the abstraction of blood is indicated, this medicine is improper. The labour should have made some progress; the parts should be well lubricated with the natural mucus; the uterine orifice fully dilated, and all the soft parts prepared for delivery. The practitioner should, by careful examination, &c. be assured that delivery is retarded only by defective action of the womb. The presentation should be natural, and the child so situated that delivery can be effected by the efforts of the uterus.*

"If the patient is very much fatigued and feeble, it should not be given till she is refreshed and recruited, by suitable nourishment or medicine, lest the exertion it occasions should be more than she can bear."

---

2. *Uterine Hæmorrhage.* Dr. Bedel has related, in a late number of the *Gazette de Santé*, a curious case of this kind, in which he was induced, all other means failing, to plug—not the vagina, but the uterus itself. The patient, after a tedious labour, and the extraction of the placenta, had lost, according to the Doctor's computation, fifteen pounds of blood—had successively fainted—and appeared in the jaws of death. In this extremity, and having exhausted all the usual means of arresting the hæmorrhage, he prepared to plug the vagina. But reflecting that, if the vessels poured forth blood internally, the patient might be destroyed by the quantity which the uterus itself would hold, he decided on plugging the latter organ from the fundus. He, therefore, intro-

"* It may be observed, that in some cases, where the pelvis was a little confined, and where the head was not sufficiently low down for the application of the common forceps, the secale has been successfully used, and the child delivered with the forceps: very great discretion, however, in these cases is required."

duced successive balls of dressed hemp (the only thing at hand) each about the size of an orange, and to the number of eight, so as to completely fill the uterine cavity. By this procedure, and by the application of pressure on the abdomen, the hæmorrhage was completely arrested, and the patient soon revived so far as to be able to speak. Dr. B. determined not to withdraw the waddings till the next day. No hæmorrhage intervened. On the following day he introduced the hand and grasped one of the tampons. By means of a hook, he contrived to extract the plug without removing the hand already in the vagina. In this way he went on, and successively, as well as successfully, withdrew the whole of the plugs. There was no more hæmorrhage, and the patient did well.

There are no comments made by M. Miguel (the editor, who is himself a noted accoucheur) but we confess that the process appears to us of very doubtful utility. It is on contraction of the uterus that all our hopes of arresting hæmorrhage depend, and this contraction cannot take place, when its cavity is stuffed with a foreign substance. We leave the case, however, to the consideration of our obstetric readers.

---

3. *Transfusion of Blood.* Mr. Waller, of Aldersgate-street, has lately performed this operation in a case of uterine hæmorrhage, the particulars of which case we now lay before our readers.

The female was of delicate strumous habit. A large quantity of liquor amnii had been discharged before our author's arrival, but there being no appearance of speedy delivery, Mr. W. left the patient, three hours after which the pains came on and the child was soon born. Mr. Jesse was then in attendance, and soon after delivery Mr. W. was sent for. He found the patient, "to all external appearance, dead." She was lying on her back completely blanched—lips bloodless—hands and feet cold—power of deglutition suspended—no apparent respiration. Some trifling pulsation, however, could be felt in the radial artery. It was found that the expulsion of the placenta had been followed by a profuse hæmorrhage, and the state of collapse above described. The proper applications had been made to restrain the hæmorrhage, but there was still a draining from the uterus. Some brandy and ammonia were exhibited internally. The power of deglutition returned—and the pulse rose a little after each administration of stimuli, but soon sunk again. The hæmorrhage ceased entirely, and the patient was getting generally cold. She was wrapped up in blankets, and the brandy continued, but with no better success. Dr. Blundell was now summoned, and attended; but, as the pulse was perceptible, it was not judged proper to perform the operation of transfusion at that time. In an hour, the patient had rather lost than gained ground. The operation was therefore determined on, and performed in the usual manner, the blood being drawn from the patient's husband. Two ounces were injected, but no effect was apparent. Two ounces more were thrown in, and a tendency to syncope was observed, the pulse falling a little. Some effort at vomiting was also evinced. These symptoms ceased spontaneously, in a minute or two. The patient expressed herself, as easy, and no more blood was injected. The pulse fell from 120 to 110, but was still very feeble. In six hours the patient had rallied considerably —pulse 100 and rounder. Complained of hunger. A nourishing diet was allowed her, and she recovered without a bad symptom.

We think that this case can only go to prove that the operation may be done without disadvantage. It would be injudicious to claim more in the present instance—but this is a great point gained for the proposers of the transfusion.

Since the above, another case, less equivocal, has occurred in the practice of Mr. Doubleday, where a female was on the point of death, from uterine hæmorrhage, both before and after the separation of the placenta. The poor woman being nearly moribund, Dr. Blundell was called in, and advised the operation of transfusion. It was objected to, but afterwards Mr. D. himself performed it, and evidently with the best effects. The patient appeared to be snatched from the jaws of death by the transfusion of 14 ounces of blood.—*See Med. and Phys. Journal for November* 1825.

A third case has recently occurred, in the practice of Mr. Wright, and under the eye of Dr. Uwins and some other medical gentlemen. The transfusion was had recourse to, as a dernier resort, and there is every reason to believe that life was preserved by the operation. Upon the whole, we are disposed to view transfusion in a favourable light, when carefully performed, and under circumstances where all other means have failed.

---

4. *Extra-uterine Conception.* A case of this kind lately led to the operation of gastrotomy, in America, but without success. Dr. Wishart, of Washington, and Dr. Stevens examined the uterus and abdomen of a woman, and agreed that there was an extra-uterine conception. They proposed an operation, and the woman consented. Dr. Wishart made an incision on the right side, an inch from the umbilicus, in the direction of the linea alba, and six inches in length. On cutting through the peritoneum and membranes, a considerable quantity of water was discharged, and one of the legs of the fœtus presented, and was laid hold of. The head was in the left iliac region. The membranes had formed considerable adhesions to parts in contact, but were, it is said, easily separated. The placenta and portion of membranes attached to it, were partially lodged in the cavity of the right fallopian tube. The wound was closed with sutures, adhesive straps, and a bandage. The fœtus weighed one pound seven ounces—the placenta two pounds five ounces. Our author being obliged to leave home, did not see the patient till the fifth day from the operation, when the abdomen was much swollen—in short, she was at the point of death.

On examination, the whole surface of the peritoneum presented marks of inflammation approaching to gangrene. The right fallopian tube was much diseased. The uterus was healthy.—*Philadelphia Journal, No. 1. New Series.*

Considering that the woman was "able to follow her common business" up to the time of the operation, and bearing in mind, that many women have carried extra-uterine conceptions for a number of years, with no great inconvenience, we confess that we should hardly have recommended so severe and doubtful a remedy, under the then existing circumstances. We do not say that the operation was *unjustifiable*—far from it; but a thing may not be *unjust*, and yet not quite *judicious*. We believe there are more instances on record of life *maintained*, under the unfortunate accident of extra-uterine conception, than of life *preserved* by the operation of gastrotomy.

---

5. *Vesico-vaginal Apertures.* A more deplorable accident cannot happen in the parturient state than the formation of an aperture between the neck of the bladder and the vagina. The constant percolation of urine through the opening renders life a dreadful punishment, and it is hardly possible for the practitioner to escape from the censure of misconduct or ignorance. Whether it is possible, in all cases, to avoid this accident, is a question which we shall not discuss, but certain it is that a remedy for the evil, when it does occur, is a desideratum in surgery. Various plans have been proposed for the cure of these apertures, and in some instances, as our readers know, success has been obtained.

Professor Lallemand, of Montpellier, has lately published the details of a case where, from the ignorance and obstinacy of a midwife, the neck of the bladder sloughed, from long pressure of the head, and a transverse aperture, of considerable extent, was the consequence. When the Professor examined the case, more than two months after the delivery, he found the edges of the aperture callous, the opening being six or seven lines in extent. The poor lady was in a dreadful condition, being obliged to keep almost constantly in the recumbent posture, with a catheter in the bladder. The indications which he proposed to fulfil were, to cauterise, with lunar caustic, the callous edges of the aperture, and, when the sloughs fell off, to bring the inflamed and suppurating edges in contact, and there to retain them till adhesion took place.

We cannot convey to our readers any clear idea of the instrument which he had constructed for the retention of the lips, without the plates which are contained in the *Archives Generales for April* 1825. The caustic was applied easily by means of a canula, but the other instrument was of very complicated construction, and kept the parts in apposition, by means of crooked needles, in a way somewhat analogous to the operation for the harelip. He failed in the first attempt, at least partially; but in the second, the adhesion was completed, and the lady was restored to a comfortable enjoyment of her existence.

It is hardly necessary to say, that during the application of the instrument for the coaptation of the sides of the aperture, it was necessary to keep a catheter in the bladder, in order to prevent the percolation of urine through the opening.

